# Sleep-Based Interventions in Alzheimer’s Disease: Promising Approaches from Prevention to Treatment along the Disease Trajectory

**DOI:** 10.3390/ph14040383

**Published:** 2021-04-19

**Authors:** Susanna Cordone, Serena Scarpelli, Valentina Alfonsi, Luigi De Gennaro, Maurizio Gorgoni

**Affiliations:** 1 UniCamillus, Saint Camillus International University of Health Sciences, 00131 Rome, Italy; susannacordone@gmail.com; 2 Department of Psychology, University of Rome “Sapienza”, 00185 Rome, Italy; serena.scarpelli@uniroma1.it (S.S.); maurizio.gorgoni@uniroma1.it (M.G.); 3 IRCCS Fondazione Santa Lucia, 00179 Rome, Italy; valentina.alfonsi@uniroma1.it

**Keywords:** Alzheimer’s disease, sleep, obstructive sleep apnea syndrome, continuous positive air pressure, sleep-based interventions, NREM sleep, SWS enhancement

## Abstract

The multifactorial nature of Alzheimer’s disease (AD) has led scientific researchers to focus on the modifiable and treatable risk factors of AD. Sleep fits into this context, given the bidirectional relationship with AD confirmed by several studies over the last years. Sleep disorders appear at an early stage of AD and continue throughout the entire course of the pathology. Specifically, sleep abnormalities, such as more fragmented sleep, increase in time of awakenings, worsening of sleep quality and primary sleep disorders raise with the severity and progression of AD. Intervening on sleep, therefore, means acting both with prevention strategies in the pre-clinical phase and with treatments during the course of the disease. This review explores sleep disturbances in the different stages of AD, starting from the pre-clinical stage. Particular attention is given to the empirical evidence investigating obstructive sleep apnea (OSA) disorder and the mechanisms overlapping and sharing with AD. Next, we discuss sleep-based intervention strategies in the healthy elderly population, mild cognitive impairment (MCI) and AD patients. We mention interventions related to behavioral strategies, combination therapies, and bright light therapy, leaving extensive space for new and raising evidence on continuous positive air pressure (CPAP) treatment effectiveness. Finally, we clarify the role of NREM sleep across the AD trajectory and consider the most recent studies based on the promising results of NREM sleep enhancement, which use innovative experimental designs and techniques.

## 1. Introduction

Alzheimer’s disease (AD) affects at least 27 million people, and its occurrence has a dramatic impact on the life of patients and caregivers, in addition to elevated financial costs to society [1].

Sleep disorders and alterations of macro- and microstructural sleep features are common in the elderly population [2,3,4]. Some sleep problems overlap in healthy aging and dementia, and a great deal of empirical evidence shows that sleep alterations among these groups of subjects are similar but are more pronounced in patients with AD [5]. It is worth mentioning that sleep disturbances are typical of other forms of dementia [6], and some autopsy studies have shown that AD and dementia with Lewy bodies (DLB) pathologies (the second cause of dementia) frequently co-occur [7,8]. DLB is mainly characterized by patients showing a high percentage of disturbances of movement and control during sleep, such as restless leg syndrome (RLS), periodic limb movements (PLMS) and sleepwalking (SW) [9]. New methodological perspectives derive from advances in AD pathogenesis, with clinical signs observable many years before the onset of cognitive deficits. For this reason, research on healthy elderly individuals has become crucial to follow the evolution of the disease over time, to detect early markers of the disease, and to develop early therapeutic interventions that could slow down the progression of AD and act promptly on the collapse of cognitive functions.

In this light, sleep represents a modifiable and treatable risk factor for AD. The perspectives and methodologies of research on the relationship between sleep and AD have changed over time [10]. In the last ten years, several areas of research have brought to light new knowledge that relates sleep abnormalities and AD aiming to identify possible early markers of neurodegenerative disease among the various features of sleep. The number of longitudinal studies in healthy elderly subjects, Mild Cognitive Impairment (MCI) and AD is recently growing. It has become crucial to monitor sleep of healthy elderly subjects over time to draw a continuous line of sleep-related changes in association with cognitive decline and the known markers of AD pathogenesis, such as beta amyloid (Aβ) and tau protein.

This review considers, at first, sleep abnormalities and primary sleep disorders, in particular insomnia, sleep breathing disturbances (SBD), circadian rhythm disturbances, and excessive daytime sleepiness (EDS), in qualitative and quantitative terms to provide a comprehensive view of the most recent advances associating poor sleep quality and AD. It contextualizes the main results of these studies in the light of new findings on pathophysiological mechanisms associating sleep with AD. Empirical evidence underlining the well-known relationship between sleep disturbances and AD/MCI condition is also mentioned. Then, it focuses on evidence that encloses sleep and AD along a trajectory, starting from normal aging and crosses the evolution of the disease, providing a comprehensive view of the current state of the art. With this aim, we consider mainly longitudinal studies that investigate sleep pathway in predictive and evolving terms in relation to AD.

Furthermore, we emphasize the role of obstructive sleep apnea (OSA) disorder, given the growing evidence of its overlapping and sharing mechanisms with AD. OSA could be viewed as a representative condition of the relationship between sleep and AD due to its main features (hypoxia, sleep fragmentation, changes in sleep structure, and adverse consequences on the brain).

Then, we explore sleep-based intervention, considering AD as a continuum that starts from the pre-clinical stage and follows the trajectory of the disease, suggesting that any intervention strategy should be administered as early as possible to achieve effective results to improve patients’ quality of life and slow disease progression.

After an update on sleep hygiene-based interventions, combination therapies, and bright light therapy (BLT), we provide a new in-depth look at the continuous positive air pressure (CPAP) as a possible new target therapy, in the light of the close relationship between OSA and AD.

Finally, we focus on the current knowledge on the specific role of NREM sleep alterations in the trajectory of AD. We review the most recent studies based on promising results on NREM sleep enhancement as a possible strategy to prevent and/or slow down the AD pathology, using innovative experimental designs and techniques to provide a comprehensive view of the most promising therapeutic approaches.

## 2. Sleep Disorders across Alzheimer’s Disease Progression

Approximately 60% of AD patients exhibit sleep disturbances that occur from the pre-clinical stages of the disease [11]. Sleep changes in AD patients include difficulty falling asleep, early morning awakenings, and daytime sleepiness, as well as primary sleep disturbances such as insomnia, SDB, circadian rhythm disturbances and EDS [12]. Mild and moderate AD patients show signs of sleep fragmentation, expressed as an increase in the number and duration of awakenings, reduced slow-wave sleep (SWS) and REM sleep, increased N1 sleep, and shorter sleep duration [13]. Regarding circadian rhythm dysfunctions, patients complain of sundowning, expressed by a state of nocturnal agitation and anxiety that resolves during the day, but constitutes a factor of worsening quality of life of both patients and caregivers [14].

A high percentage of MCI patients ranging from 8.8% to 45% also suffer from sleep disturbance, similarly but less markedly than AD patients. Many studies found less time spent in REM sleep, a lower amount of SWS in MCI compared to healthy elderly. Furthermore, insomnia, SBD, circadian rhythm disturbances and EDS are also present in MCI patients [15].

About twenty years ago, more in-depth investigations of the relationship between sleep disorders/alterations and cognitive decline began in populations of healthy older adults. The rationale was based on the evidence that sleep disorders occur at very early stages of AD. The limitation of these studies is determined by the fact that most of them were based on subjective assessments of sleep quality provided by patients or caregivers, which are poorly reliable. Objective measures obtained through actigraphy and polysomnography (PSG) allow greater reliability, and their importance, also in the field of dementia, should be carefully considered by the scientific community. The PSG is not commonly included in the clinical assessment of AD patients. On the other hand, PSG studies in AD/MCI are recently growing, allowing a more extensive knowledge about the relationship between sleep alterations and AD pathophysiology (see Section 4).

Insomnia is common in AD and has been extensively studied in relation to cognitive decline in healthy elderly subjects. However, the results are still heterogeneous. A longitudinal study with a 3-year follow-up in an elderly sample showed that self-reported chronic insomnia independently predicted the risk of cognitive decline, but only in men [16]. Another longitudinal study did not confirm this finding but found that subjects who exhibited EDS at baseline were twice as likely to develop cognitive impairment [17]. Jaussent and colleagues [18] conducted another longitudinal study with 8-year follow-up [18] examining the association between different insomnia symptoms, EDS, as assessed by questionnaires at baseline, and cognitive decline in healthy older adults. Results showed that EDS was significantly associated with a 30% increased risk of developing cognitive decline. The number of insomnia complaints and intra-sleep awakenings was negatively related to cognitive decline. The effects were specifically observed in subjects with cognitive decline, which had developed dementia during the follow-up period. Authors suggest that the presence of EDS in older people with some obvious symptoms of cognitive decline could be symptomatic of an early stage of brain injury in areas that might initially cause abnormalities in sleep and/or the circadian system [18].

At the same period, a longitudinal study on 2012 healthy elderly with follow-up at 2 and 10 years [19] showed that daytime napping, nighttime sleep duration and EDS could represent modifiable risk factors or clinical indicators of future development of cognitive decline. Despite empirical evidence correlating specific parameters related to sleep quality with subsequent cognitive decline, some other findings are not coherent. For example, in a longitudinal study with follow-up after two years from baseline, Tworoger and colleagues [20] found, in a population of older women, that cognitive impairment related to short sleep duration and difficulty falling asleep was present only at baseline but no differences were observed after two years. 

Given the limitations of self-reported measurements regarding sleep quality and quantity, some studies have investigated the relationship between disturbed sleep and cognitive decline using actigraphy as an objective method to investigate sleep. In particular, a longitudinal study [21] investigated the associations of both objectively and subjectively sleep measures with subsequent cognitive decline. Actigraphic data showed that higher levels of intra-sleep awakenings, prolonged wakefulness episodes, and lower sleep efficiency were associated with a significantly increased risk of developing cognitive decline, with particular reference to executive functions. Another prospective study with 6-year follow-up and objective measurement of sleep by actigraphy (ten consecutive days) in non-demented elderly subjects found that greater sleep fragmentation was associated with greater risk of developing AD [22]. An association between high day-to-day variability in actigraphically-determined sleep duration and lower subjective well-being was noted in a cross-sectional work [23] conducted in a healthy sample. In contrast, sleep duration, falling asleep latency, and intra-sleep awakenings were not related to subjective well-being. Furthermore, the relationship between variability in sleep duration and well-being was partially mediated by subjective sleep quality. Thus, large variability in sleep duration correlated with subjectively perceived poor sleep quality and low well-being. 

Regarding sleep duration, many cross-sectional studies have emphasized a U-shaped relationship between self-reported sleep duration and cognition, as both too long and too short sleep durations were associated with worsening cognitive function [24,25].

Starting from the above-mentioned associations that link poor sleep quality with AD, investigators have hypothesized several possible mechanisms underlying this relationship, assuming both pathological processes common to sleep and AD and a mutual influence between them. These mechanisms include structural and functional changes in some brain regions related to sleep and cognition (in particular, the areas of the default mode network—DMN), increased neuronal excitability, oxidative stress, abnormal pattern of glucose metabolism, defects in the clearance of toxic substances in the brain (especially Aβ), neuronal loss (with particular reference to locus coeruleus noradrenergic neurons).

The involvement of DMN in relation to sleep and AD is crucial in light of the new advancement in the pathomechanisms of this relationship. A cross-sectional study, based on MRI [26], investigated the relationship between chronic daytime sleepiness and connectivity in six brain networks in healthy young and elderly subjects. Elderly subjects had less self-perceived daytime sleepiness than young subjects and showed reduced connectivity in the DMN. The main areas of the DMN were the hippocampus, para-hippocampal gyrus, medial prefrontal cortex, lateral temporal and temporo-parietal regions, and medial posterior cortices (posterior cingulate cortex and precuneus). This decreased connectivity in DMN structures could reflect a kind of “local sleep” phenomenon, where parts of the brain expresses a sleep state during wakefulness [26].

It is interesting to note that fragmentation is one of the main and common characteristics of sleep-related disorders: insomnia, breathing disorders, intra-sleep awakenings, poor sleep quality, EDS, sleep-wake rhythm disorders are based on a fragmented sleep that, as evidenced by many studies carried out with sleep deprivation protocols [27,28], have adverse effects on cognition, behavior and, in general, on the quality of life. Indeed, sleep deprivation appears to contribute to Aβ deposition. Soluble Aβ is released during physiological synaptic activity, which is greater during wakefulness, as well as the release of Aβ. During sleep, however, neuronal activity and Aβ levels decrease. Notably, the regions of the DMN are the ones that exhibit the greatest activity in wakefulness and the highest amount of Aβ in the AD development [29]. Therefore, it is possible to hypothesize that low sleep quality and duration could enhance neuronal activity and the related excess of Aβ deposition, increasing the risk of accumulation and subsequent aggregation in amyloid plaques, especially in areas that show increased neuronal activity. This mechanism raises the question regarding possible pathogenetic mechanisms between sleep and AD, highlighted by the specificity of DMN structures common to sleep alterations and AD.

Sleep fragmentation means to interrupt the continuity of regular sleep, and this phenomenon has important implications: decreased slow wave sleep (SWS) and REM sleep, subsequent daytime sleepiness, metabolic changes, increased oxidative stress, increased neuronal and synaptic excitability, misalignment of homeostatic and circadian rhythms. Regarding SWS, its amount represents the best indicator of reduced Aβ levels, suggesting that reduced or fragmented SWS might initially increase soluble Aβ levels and subsequently facilitate the deposition and formation of Aβ plaques in the brain [30]. The cingulate gyrus appears to contribute to the propagation of slow waves to other areas, such as the precuneus [31]. Therefore, it is possible to hypothesize that a deteriorated connectivity as a consequence of increased Aβ accumulation in the cingulate gyrus could generate greater sleep instability and reduced amount of SWS.

Recent animal studies have also attributed a role of sleep in the clearance of toxic substances (including Aβ) in the brain by the glymphatic system [32,33], a perivascular network supporting CSF-interstitial fluid exchange. The close association between sleep and glymphatic system is particularly evident in the elderly and in neurodegenerative diseases. Sleep characteristics of these populations, such as disruption of sleep architecture, increased sleep fragmentation, sleep disturbances and decreased time spent in deep sleep stages (N3), could cause a dramatic decrease in Aβ clearance. In fact, Aβ clearance and glymphatic fluid transport efficiency positively correlate with the amount of SWS (for a review, see [34]).

The glymphatic system appears to be regulated by the sleep-wake rhythm, since more movement of CSF tracers through brain tissues have been found in sleeping rats than in awake rats [32]. Beneficial decreases of Aβ during sleep appear to be the product of lower synaptic activity during this state, as well as the decrement in the synaptic release of Aβ and increased glymphatic system clearance of extracellular Aβ during NREM sleep [35].

In the context of the most common sleep disturbances in healthy elderly, MCI, and AD patients, the issue of changes in circadian rhythms is of particular interest, especially for the successive intervention strategies that are often based on the regulation of sleep-wake rhythms. Empirical evidence shows poor consolidation of rest-activity rhythms, reduced amplitude of circadian rhythms, and misalignment of circadian markers such as body temperature and cortisol levels in AD patients [36]. Specifically, approximately 40% of AD patients complain severe dysfunctions of circadian systems with manifestations that include daytime sleepiness, sundowning, agitation, and day-night reversal [37,38]. This clinical symptomatology is consistent with neuronal degeneration of the suprachiasmatic nucleus of the hypothalamus, the main structure regulating circadian pacemakers, and decreasing melatonin levels in the CSF starting from the pre-clinical stages of AD [36].

Tranah and colleagues [39] first investigated the relationship between circadian activity rhythms and subsequent development of MCI and dementia in a population of healthy older women. After a follow-up of approximately 5 years, 15% of the women had developed dementia, and 24% had become MCI. An increased risk of dementia or MCI was found after approximately five years in healthy women with decreased circadian rhythm amplitude and strength and delayed rhythms. These associations were independent of sleep fragmentation and sleep duration.

A cross-sectional study showed that the MCI group exhibited an advanced timing of onset of melatonin secretion compared with the control group, without differences in the melatonin levels [36]. Regarding sleep structure, differences were observed in terms of increased wakefulness after sleep onset and increased latency of REM sleep in the MCI group. Earlier onset of melatonin secretion also correlated with worse performance on memory tasks in the MCI group. Thus, circadian misalignment and sleep disruption were evident in MCI patients. This was the first study to concurrently assess salivary melatonin and sleep measured by PSG in MCI patients. The authors proposed a deterioration of common neurobiological circuits related to sleep and cognitive function. Indeed, the circadian timing observed in MCIs correlated with a reduced ability to form new episodic memories during the evening.

Concerning neurotransmitters, it is possible that the noradrenergic system is implicated, particularly with regard to its role in pineal melatonin synthesis [40]. The cholinergic system, which projects from the basal forebrain to the hypothalamus, has received much attention [41]. This circuit appears to be critical for memory, REM sleep onset, wakefulness and arousal regulation [41,42]. Atrophy of the basal forebrain has previously been observed in both MCI and AD [43], and the integrity of associated fiber tracts from this region may be compromised [44].

Gabelle and coworkers [45] aimed at identifying self-reported disorders related to the sleep-wake cycle that increase the risk of cognitive decline after 1-year follow-up in subjects considered frail at risk of cognitive decline. Frailty was assessed considering the presence of adequate functional status, and a criterion of frailty based on memory impairment, limitations in one of the instrumental activities of daily living or a slow gait in walking. Results showed that subjects with EDS had an increased risk of cognitive decline assessed by the MMSE. Besides, longer time spent in bed was associated with cognitive decline. Patients with a decline in MMSE and EDS scores showed more napping, more REM sleep disturbance, fatigue, and insomnia. Those who spent more time in bed, on the other hand, showed longer total sleep time over 24 h but with longer intra-sleep wakefulness.

### An Emerging Strict Relationship: OSA and AD

In the context of the relationship between sleep abnormalities and AD, OSA needs to be treated in more depth, given the growing amount of new research that closely associates OSA and AD, even hypothesizing possible shared and mutually affecting mechanisms between these two conditions. AD patients are five times more likely to have OSA when compared with controls, and OSA appears in approximately 50% of AD patients [46]. Furthermore, OSA could be considered as a possible undiagnosed and underlying cause of sleep disturbance, such as insomnia, EDS, sleep fragmentation, arousal and nocturia. The underestimation of OSA condition in the clinical practice could also be due to the fact that OSA patients do not notice their own apnea episodes during the night and it is often their bed partners who report the presence of such episodes. Although such evidence does not imply directionality or causality per se, it does emphasize the importance of the relationship between OSA and AD. Indeed, the main features of OSA, such as hypoxia, sleep fragmentation, changes in sleep structure and consequences on the brain certainly represent the most prominent picture of the relationship between disturbed sleep and AD. 

AD and OSA share several risk factors such as age, gender, cardiovascular comorbidities, and genetic background [47]. In particular, OSA is primarily a vascular risk factor, and it is well known that cerebrovascular disease is associated with worsening in cognitive performance of AD patients [48]. In fact, autoptic studies reveal that a high percentage of AD cases have a combination of AD pathology and vascular pathology, suggesting a close and reciprocal relationship between AD and cerebrovascular disease [49].

Recent evidence shows that OSA is associated with a treatable form of dementia known as Idiopathic normal-pressure hydrocephalus (iNPH) characterized by intracranial venous hypertension delaying drainage of CSF into the cerebral venous sinuses [50]. The rationale is that the respiratory efforts during sleep apnoea episodes increase the negative intrathoracic pressure, leading to a decrease in venous return to the heart ensuing in retrograde intracranial venous hypertension. Furthermore, intra-sleep awakenings worsen CSF-ISF exchange, contributing to hydrocephalus [50]. The CSF drainage significantly improves cognitive performance in patients with CSF constellations typical for AD when compared with a group without CSF AD signs, suggesting an association between AD and NPH and a possible dichotomy of a neurodegenerative NPH and a true idiopathic NPH [51]. 

Also, a number of pathological mechanisms associated with the bidirectional relationship between disturbed sleep and AD seem to be very blatant in the case of OSA. The results of a study evaluating the relationship between EDS and Aβ accumulation [52] showed that informant-reported apnea (witnessed apnea) was the only sleep-related symptom that differed between participants with and without EDS. It suggests that there is a higher prevalence of OSA in individuals with EDS. Other studies show that sleep fragmentation with increased N1 and the corresponding decrease in SWS appear to be widely associated with sleepiness in OSA patients [53,54,55,56,57]. Furthermore, a study of 1552 participants (patients with cognitive decline and healthy control subjects), investigating the comparison of subjective sleep characteristics and objective sleep characteristics measured by PSG, showed that only indices related to SBD, after adjustment for confounding factors, were independently associated with a worsening in cognitive performance. It was hypothesized that breath disturbances represented the cause of sleep structure changes in terms of fragmentation, changes in architecture, and subsequent daytime sleepiness [58].

In this view, OSA seems to involve all (and more) of the above mechanisms regarding the consequences of fragmented or disturbed sleep on the brain. OSA is widely linked to oxidative stress, glucose homeostasis, decreased connectivity in brain areas related to cognition, loss of noradrenergic neurons in the locus coeruleus, deficit in the clearance of toxic substances in the brain, and gray matter abnormalities.

Regarding the potential mechanisms that could underlie the relationship between OSA and AD, the work of Yaffe and coworkers [59] aimed to determine the prospective relationship between SDB and cognitive impairment in a population of 298 cognitively healthy elderly women with OSA and a non-OSA control group, with follow-up after 4.7 years. Compared with a control group, women with OSA were more likely to develop MCI or dementia. Specifically, a high oxygen desaturation index and a high percentage of time spent in apnea or hypopnea during sleep were associated with the risk of developing MCI or dementia. In contrast, measures related to sleep fragmentation (arousal index and intra-sleep awakenings) or duration were not associated with cognitive decline development. Sleep time spent with oxygen saturation <90 was not associated with prospective cognitive decline. These results suggest that intermittent hypoxemia is gravely harmful to the development of cognitive decline compared with continuous hypoxemia. The absence of association between sleep fragmentation and duration and cognitive impairment suggests that the mechanism associated with worsening cognitive function is mediated primarily by hypoxemia.

Therefore, recent studies have investigated the relationship between OSA and AD, trying to correlate Aβ deposition with different levels of OSA severity. A recent study [60] showed that plasma Aβ 1–40 concentrations in a group of patients with severe OSA were significantly greater than in groups with lower OSA severity. This finding seems to be related to the severity of hypoxia, which could indicate an increased risk of developing AD in this group of patients. The correlation between serological levels of Aβ 1–40 and hypoxia during sleep would, therefore, be quantitative: the higher the levels of hypoxia, the higher the concentration of Aβ 1–40 in plasma. Moreover, the greater concentration of Aβ 1–40 correlates with apnea-hypopnea index (AHI) and, negatively, with SpO2. 

Recently, the first systematic review by Fiedorczuk and co-workers [61] reported a qualitative evaluation of observational analytic studies to establish whether there is an association between OSA and oxidative stress. While oxidative stress was associated with OSA, no clear difference in the level of oxidative stress has been found between OSA patients with or without cardiovascular complications. Whatever the cause of oxidative stress, it is associated with adverse effects on cell membranes, DNA structure, and proteins, causing conformational changes and damage. Intermittent hypoxia, fragmented sleep, and disturbances in breathing would be the cause of oxidative stress and fluctuations in cerebral blood flow that could induce edema [62,63], leading to gray matter hypertrophy that is considered a possible early sign of neurodegeneration in OSA and could evolve, if left untreated, and progress leading to atrophy. In contrast, OSA has also been associated with gray matter decrement in the hippocampus, cingulate, and cerebellum, as well as in the temporal, frontal, and parietal lobes [64,65].

Damage produced by oxidative stress is also part of the pathogenesis of cognitive decline associated with both aging and neurodegenerative disorders. In humans, hypoxemia seen in OSA has been associated with a delay in peripheral nerve conduction [66] and dysfunction of the blood-brain barrier, due to alterations in microvascular permeability also induced by oxidative stress [67].

Another recent review [47] analyzed the literature regarding OSA and neurodegeneration. The results of several prospective studies showed that individuals with OSA at baseline were more likely to develop cognitive decline and, consequently, evolve into dementia over the years. OSA has also been associated with a defect in the process of Aβ clearance. Indeed, continuous sleep fragmentation could alter the homeostasis of CSF-ISF exchange and predispose to an increase in Aβ accumulation. In particular, intermittent hypoxia and OSA-related hemodynamic changes could cause abnormalities in water and solute fluxes within the brain and contribute to dysregulation of the glymphatic system [68,69].

Observations regarding the association between OSA and glucose metabolism are of interest because glucose homeostasis and insulin regulation are recognized as risk factors for cognitive decline [70]. In general, higher fasting blood glucose and reduced metabolism are linked to poor cognitive performance and brain atrophy [71]. Given the high proportion of OSA patients with insulin resistance or diabetes mellitus (15% to 30%), it is possible to speculate that there is a relationship and that the effects of OSA on glucose metabolism could also contribute to cognitive decline.

OSA also appears to contribute to a reduction of the noradrenergic neuronal population in the locus coeruleus [72]. The locus coeruleus is a brain area implicated in synaptic plasticity, motor control, and other functions related to cognitive decline in the elderly. Studies have shown that lower neuronal density and decreased connectivity in the locus coeruleus is associated with lower cognition at baseline, MCI and faster cognitive decline. In addition, studies with functional imaging found that OSA decreased brain activation (and a parallel decreased connectivity) in cingulate, frontal, and parietal regions during memory and sustained vigilance tasks [46]. 

An interesting work by Bubu and collaborators [73] examined the relationship between OSA and longitudinal changes in brain amyloid PET deposition and CSF biomarkers. The mean follow-up of this longitudinal study was 2.5 years. The innovation of this study concerns the question of determining whether the OSA-AD association is related to AD neuropathology changes over time. Relationships between longitudinal changes in biomarkers related to Aβ and tau protein and the clinical diagnosis of self-reported OSA in a sample of cognitively healthy, MCI, and AD subjects were assessed to provide further confirmation for a possible causal relationship between OSA and AD. The results pointed to significant differences in the annual rate of change in florbetapir uptake, Aβ42 CSF, T-tau, and P-tau along the follow-up period for the healthy and MCI groups, with OSA+ subjects having a significantly more rapid increase in amyloid and aggregated tau levels. The authors hypothesized several mechanisms that could be responsible for these findings: intermittent hypoxia, sleep fragmentation, fluctuations in intrathoracic and intracranial pressure, and increased venous pressure, which would act as an impediment for the circulation of brain metabolites from interstitial fluid to CSF via the glymphatic system, leading to Aβ accumulation [74].

The relationship between OSA and AD has also been recently studied specifically in relation to tau protein levels by considering some regions of interest as highly susceptible to tau accumulation (i.e., the entorhinal and inferior temporal cortices). Results in elderly subjects without cognitive impairment showed that those with witnessed apnea had higher tau levels in the inferior entorhinal and temporal cortex [75].

A cross-sectional study of 2909 cognitively healthy older men [76] showed that the severe degree—but not the mild degree—of nocturnal hypoxemia was associated with poor cognitive performance. Time spent in REM sleep also correlated with poor cognitive functioning, particularly in the executive function and attention domains. The higher percentage of time spent in stage 1 was also associated with the same cognitive domains but also with global cognitive status. Hypoxemia has many adverse effects on the brain, associated with several conditions that, in turn, play a role in the AD pathogenesis. In fact, an animal study showed that experimentally induced hypoxemia led to cholinergic damage, inflammation, and oxidative stress, and all of these effects have a relationship to the pathogenesis of dementia and deficits in spatial memory and attention [26]. Thus, the results of this study support the hypothesis that hypoxemia plays a role in the pathophysiology of cognitive deficits associated with SDB.

## 3. Sleep-Based Intervention Strategies

To date, there are no therapies for the treatment of AD: all clinical interventions are aimed at slowing the disease progression and improving the patients’ quality of life. In view of a neurodegenerative disorder that seems to have a multifactorial nature, researchers are giving growing importance to the identification of risk and modifiable factors for the prevention and treatment of AD and AD-related comorbidities.

In recent years, sleep fits very strongly into this context, although sleep-based interventions are still few and poorly structured. This is partially due to the fact that sleep assessment and monitoring are not yet included in the clinical routine related to AD and cognitive impairment, leading to a general underestimation of sleep-related problems in AD patients. Some sleep-related treatments have been studied and found to be effective in both the elderly and patients with cognitive decline and AD.

In this section, we summarize the well-known sleep-based intervention strategies concerning the use of sleep hygiene, combined interventions and BLT. Then, we focus on Continuous Positive Air Pressure (CPAP), highlighting the concept that each of these interventions should be administered early to prevent or slow down as much as possible the course of AD. A summary of the main results of sleep-based intervention strategies is reported in Table 1.

### 3.1. Behavioral Strategies, Combined Interventions and Bright Light Therapy

In AD patients, given the risk of possible adverse effects of sedative medications, behavioral strategies are often recommended. These strategies are based on sleep hygiene rules or combine a series of behavior designed to improve sleep quality and cognition. In general, sleep hygiene rules include regularity in bedtimes, limiting naps during the day, avoiding excitatory substances such as alcohol, nicotine, and caffeine, maintaining a room temperature that is not too hot or too cold, and reducing light and noise in the environment [77]. Behavioral strategies involve physical exercise, increased socialization or cognitive behavior therapy (CBT), often used to treat insomnia (CBT-I). The present literature suggests that all these strategies may represent promising treatment options in this field. 

For example, the effects of behavioral strategies have been studied in the elderly population. Some studies have observed that combining certain behavioral strategies positively affected disturbed sleep in elderly populations. One study had combined social activities and low-intensity physical activity, showing an increase in SWS after two weeks of treatment [78]. An intervention strategy that combined an increase in daytime physical activity and a program designed to decrease nighttime noise and sleep-disturbing practices in incontinent elderly (mean age 88.3 years) showed an increased sleep duration and decreased agitation levels after 14 weeks of treatment [79]. 

One study evaluated the effectiveness of a 4-week “Sleep Intervention Program” that included sleep compression, modified stimulus control, and sleep hygiene compared with a 4-week control intervention among healthy older adults [80]. Those who had participated in the sleep-based intervention showed significant improvements in sleep efficiency assessed by actigraphy, number of intra-sleep awakenings, and minutes awake during the night compared to the control group. These improvements decreased but remained significant at follow-up after four months. These results show the effectiveness of a brief behavioral intervention on sleep of healthy elderly people.

The issue concerning the possible beneficial effect of these interventions on cognition remains open. There are emerging and encouraging results from studies conducted on MCI patients. A 24-week structured limbs exercise program was administered to MCI patients [81]. It resulted beneficial for sleep quality and the maintaining of general cognitive function at 12 and 24 weeks follow-up, mostly concerning processing speed domain. Also, CBT-I induced significant improvement in sleep and cognition, especially for executive functioning [82], but no changes were observed in the verbal memory domain.

Bright light therapy (BLT) is a non invasive treatment based on the assumption that many individuals with sleep disorders have a disrupted sleep-wake cycle. This is related to age-dependent functional changes and to degeneration associated with the central nervous system, with particular reference to the supra-chiasmatic nucleus in the hypothalamus [83], as specified concerning sleep-wake rhythm disorders. Light is considered as the most powerful zeitgeber of the human circadian system [84] and some evidence highlighted the efficacy of BLT—delivered by light emitters—on sleep, cognition and behavior in dementia (for a review, see [84]). In general, the beneficial effect of BLT remain controversial, possibly due to the fact that many studies based on BLT protocols have been conducted in institutions, hospitals, or nursing homes. In fact, these protocols involve the administration of particular lighting conditions to which the subjects are submitted at different times and periods of the day and the lightning conditions in hospital or nursing home is often inadequate, insufficient or, in any case, not compatible with normal light-dark conditions.

A recent study [85] evaluated the effects of dynamic circadian lighting and individual light exposure on sleep, cognitive performance, and well-being in a sample of 14 cognitively intact individuals residing in a nursing home (aged 70–94 years). The experimental protocol included five weeks of circadian lighting and five weeks of conventional lighting in a counterbalanced order. The measurements of rest-activity were acquired by an accelerometer and cognitive functions and clinical conditions by questionnaires. The results showed that there were no differences between the two lighting conditions. However, the analysis of pooled data showed that those exposed to greater light exposure in the morning had more stable rest-activity and less fragmented sleep. In addition, fragmented sleep and longer sleep duration were indicative of worse cognitive performance.

Another recent study [86] analyzed the effects of 5 days of 90-min morning BLT exposure in institutionalized patients with mild to moderate cognitive decline (aged 70–93 years). Cognitive abilities and sleep quality improved significantly after treatment. Interestingly, in another work evaluating the effects of BLT on agitation in AD patients [87], BLT was not effective and even seemed to worsen behavioral symptoms. The authors hypothesized that light acted as a kind of annoying sensory stimulation that increased agitation in AD patients. However, the fact that BLT was not effective in patients with full-blown AD also suggests that it may not be effective in advanced stages of the disease, confirming the need for early interventions.

A possible alternative to BLT is blue-enriched lighting, which provides high circadian stimulation during the day. It is based on new technologies in light-emitting-diode (LED), which provides blue-enhanced light at high intensity during the day and low intensity at night. This line of research is very recent and, therefore, still needs much experimental confirmation. At present, results are not homogeneous. Figueiro and colleagues [88] demonstrated behavioral signs of reduced agitation and improved sleep and mood following four weeks of blue-enriched lighting in institutionalized AD patients. Hopkins and coworkers [89] found increased daytime activities and improved anxiety but also negative effects in terms of reduced sleep efficiency and quality, increased rest-activity rhythms and increased daytime activity in individuals without dementia. 

Recently, a research group performed two studies based on the effectiveness of a tailored lighting intervention in patients with AD and related dementias [90,91]. The first study was based on an all-day active or control lighting intervention for 14 weeks (*n* = 46) [90]. The tailored lighting intervention consisted of the administration of active lighting that provided a high circadian stimulus, while the control condition provided a low circadian stimulus, below the threshold for activation of the circadian system. Results showed that the Pittsburgh Sleep Quality Index (PSQI) scores improved after the active intervention compared to both the active baseline condition and the control condition. In addition, active intervention led to significantly greater differences between intervention and control in intraday variability. Measures of depression and agitation also showed significant improvements after the active intervention. Thus, the results indicate that a lighting intervention tailored to maximize circadian system entrainment may be useful for improving sleep, mood, and behavior in patients with AD or dementia. However, objective sleep measures showed no differences between active and control treatment and thus did not confirm the effectiveness on sleep quality as assessed by self-report measures. 

In the second study, the investigators administered the same all-day lighting intervention to 47 AD and related dementias patients for 25 weeks to assess its long-term impact on sleep, mood, and behavior [91]. The results confirm and extend those of the previous study. In addition to the improvement in subjective sleep and the decrease in levels of depression and agitation, an improvement in sleep efficiency measured by actigraphy was also observed. Taken together, these results are encouraging, as they emphasize the importance of the continuity of a sleep-based intervention, its duration, and the possible maintenance of beneficial effects over time.

Although in the present review we do not discuss the effects of drugs or supplements for the treatment of sleep disorders in AD, it is useful to mention the important advances in research on the role of melatonin and melatonin receptor agonist in the prevention of sleep disturbances. A recent review focused on the role of melatonin as a therapeutic mediator for sleep disorders and possible delirium—typical of advanced AD stages—in intensive care unit (ICU) [92]. Sleep loss, in fact, could lead to delirium symptoms that worsen at night and could predispose patients to act in dangerous ways. The results showed that melatonin administration decreases the effect of risk factors on the occurrence of delirium, improving sleep quality. Further investigations represent a promising research line to clarify and expand the beneficial role of melatonin in treating risk factors of AD, and its possible combination with the other reviewed strategies.

### 3.2. CPAP Intervention: A New Promising Target for AD Prevention and Care

As previously described, one of the most promising target of non-pharmacological strategies for AD from the early stages of the disease comes from recent studies that have analyzed the relationship between OSA and AD. Beyond the presence of cognitive impairment, CPAP is the elective treatment for OSA, given the numerous empirical confirmations of the effectiveness of this therapy on sleep, behavior, cognition, mood and daytime sleepiness of OSA patients. 

In recent years, there has been a robust increase in the number of works that have investigated the efficacy of CPAP in relation to AD, with the aim to improve patient’s quality of life and possibly slowing the progression of neurodegenerative processes in cognitively healthy elderly OSA or pre-clinical stages of AD.

The effects of CPAP on cognitive functioning were investigated in patients with mild to moderate AD (*n* = 52) [93]. The comparison between those receiving placebo treatment and those using CPAP for six weeks revealed no significant improvements in cognitive function. In contrast, the comparison within pre-post CPAP treatment showed significant improvements in the domains of verbal episodic learning, memory, and some aspects of executive functions such as cognitive flexibility and mental processing speed. The same research group continued with a further experimental phase, examining the long-term effects of the treatment in five patients who continued to use CPAP. In comparison with five AD patients who had stopped CPAP treatment weeks earlier, those who had continued to use CPAP showed lower cognitive decline, stabilization of depressive symptoms and daytime sleepiness, and significant improvement in subjective sleep quality [94]. Although this is a study with a very small sample, it suggests the potential therapeutic efficacy of long-term CPAP utilization for sleep-related, emotional, and cognitive variables in AD patients with OSA. 

The same authors developed an experimental protocol that involved alternating CPAP treatment and placebo in AD patients with OSA, examining the effects of 3 weeks of CPAP followed by three weeks of placebo followed by other three weeks of CPAP [95]. After a single night of CPAP treatment, AD patients (*n* = 52) showed a lower percentage of N1 and a higher percentage of N2. In the paired analysis, three weeks of CPAP led to a significant decrease in the number of intra-sleep awakenings, percentage of stage 1, arousal and increased stage 3. Therefore, the effectiveness of CPAP was visible immediately after a single night with a sleep deepening. Improvements were maintained for three weeks in the follow-up evaluation. 

The presence of OSA in a high percentage of AD patients also leads to the idea that breathing disorders during sleep, aggravating several conditions related to AD risk factors, could be associated with an earlier onset of cognitive decline. In this regard, a paper by Osorio et al. [96] showed that the presence of SDB was associated with an earlier age of onset of cognitive decline and CPAP treatment could delay the progression of cognitive impairment. 

The work of Liguori and collaborators [97] was the first to study cognitive performance, PSG recordings and CSF levels of Aβ, tau protein, and lactate in patients with subjective cognitive impairment divided into three groups: OSA, controls, and OSA treated with CPAP. The condition of subjects with subjective cognitive impairment is considered a sort of pre-MCI stage. OSA patients showed lower CSF Aβ42 concentration levels, higher t-tau/Aβ42 ratio levels than controls and CPAP-treated patients. In addition, OSA patients had reduced sleep quality and continuity and worse performance on memory, intelligence, and executive tests than the other two groups. In OSA patients, a relationship was observed between higher tau levels in CSF, disturbed sleep, and increased CSF lactate levels. Extracellular lactate in the brain is considered a biomarker of sleep-wake rhythm, since its concentration changes in relation to metabolic processes (i.e., decreased at the onset of sleep, increased during wakefulness) [78]. Given the association between sleep-wake rhythm dysregulation and AD, lactate can be considered a biomarker of both sleep impairment and AD [78]. Furthermore, lower levels of CSF Aβ42 in OSA patients correlated with memory impairment and nocturnal oxygen saturation parameters. In light of these findings, the authors hypothesized that OSA reduces sleep quality by producing intermittent hypoxia and holding down CSF Aβ42 levels, increases CSF lactate levels and alters cognitive performance in patients with subjective cognitive impairment, leading to pre-clinical AD and changes in neuropathological biomarkers. Importantly, both controls and OSA patients treated with CPAP did not show clinical or biochemical signs of AD markers, suggesting that the damage produced by OSA, especially if detected and treated early, may be modifiable. Therefore, CPAP may represent an important target of intervention also for AD. 

The effects of CPAP on sleep were reviewed by Brillante and coworkers [98] with the aim to identify and estimate predictors of SWS and REM sleep rebound after CPAP. The authors analyzed studies that included a pressure titration study protocol. Specifically, this procedure provides patients with the minimum effective pressure to eliminate all adverse respiratory events. Results showed a 40% rebound in SWS and 20% rebound in REM after the titration protocol compared with the control PSG night predicted by abnormal sleep architecture and sleep fragmentation before treatment initiation. The greater rebound in SWS is consistent with the fact that, compared to other sleep stages, SWS rebound tends to occur in the early recovery sleep period [98]. This type of measure may be useful in clinical and research practice to identify which patients might have the best effects on sleep quality after CPAP treatment: those who do not show significant SWS and REM rebound should be further monitored to control for compliance with therapy. 

The ability of CPAP to slow down the process of cognitive decline has also been investigated in another work [99]. As expressed by the annual MMSE score, cognitive decline slowed down in CPAP-treated patients when compared with untreated patients over three years. This is an encouraging result, but further studies investigating more comprehensive test batteries to assess individual cognitive domains over time are needed. 

A general examination of the literature regarding the effects of CPAP on non-demented OSA individuals reveals that the effectiveness of CPAP is related to cognition, such as memory, verbal fluency, executive functions, decreased sleep destruction and, in particular, alertness [100,101]. Furthermore, CPAP has a positive effect on factors affecting cardiac function, such as vascular resistance, coagulation and other aspects of vascular health [100]. There is much evidence confirming the efficacy of CPAP but the effects on neurocognitive function cannot yet be considered stable or complete due to the variability of outcomes (some cognitive domains appear to improve, others have no positive effect after CPAP) owing to many factors including lack of statistical power, different levels of OSA severity, and different durations of treatment [46]. 

An interesting multicenter study called The Apnea Positive Pressure Long-term Efficacy Study (APPLES) evaluated the neurocognitive effects of CPAP on OSA patients by comparing active treatment with a sham condition [102]. Results showed significant differences between the two conditions for variables related to executive and frontal functions after two months of CPAP. In contrast, there were no differences for other cognitive functions after two months or after six months. When the sample was stratified on the basis of OSA severity according to parameters related to AHI and oxygen saturation, improvements in cognition, although slight and transient, were observed in patients with severe OSA. In addition, CPAP also had a beneficial effect on daytime sleepiness: OSA patients who had received the treatment showed a greater ability to stay awake, both subjectively and objectively measured, than those who had undergone the sham condition. 

Improvement in daytime sleepiness was also observed by Rosenzweig and coworkers [103] that combined one-month CPAP treatment with psychoeducation and lifestyle modification in patients with moderate and severe OSA. This combined intervention also enhanced performance in the episodic verbal memory domain. These data are consistent with a recovery in gray matter regions and cognition in patients with OSA reported in other studies [64].

Several studies assessed the effect of CPAP on cognitive task-related local brain activity using fMRI. A longitudinal study [104] conducted on 17 OSA patients and 15 healthy control participants investigated whether the group of OSA never treated with positive air pressure (PAP) showed differences in brain activation compared to healthy control subjects during a working memory task and whether any improvement after PAP in cognitive functioning reflected a change in underlying brain activity. The fMRI scans obtained during the task were then repeated, along with cognitive and clinical assessment after three months of PAP treatment. Results at baseline showed that OSA patients had increased activation in the left frontal cortex, medial precuneus, and hippocampus and decreased activation in the caudal pons. After treatment, OSA patients showed decreased activation in the left inferior frontal gyrus and the anterior cingulate cortex and bilaterally in the hippocampus. At the same time, improvements in many neurocognitive domains were observed after treatment. This study confirms a neural compensation mechanism in never-treated patients that is reduced following effective treatment, as evidenced by improved cognitive functioning.

Interestingly, the brain of OSA patients seems to behave in a similar way to that of elderly subjects or with AD: in fact, the pattern of over-recruitment of brain regions to support an adequate level of cognitive performance is similar [105,106]. This is a further element that brings closer, even at the level of brain activity, the conditions of OSA and AD and, in perspective, the possible effectiveness of CPAP also for patients with cognitive decline or at risk of AD. 

However, the results are not homogeneous. As an example, in another work 27 untreated patients with severe OSA and 24 control subjects underwent MRI at baseline and after six months of continuous CPAP treatment [107]. No modifications in gray matter volume were observed, and there were no differences in volumes in the bilateral hippocampus and temporal lobe. No changes in gray matter density or regional volumes were observed after treatment, except for a slight decrease in total brain volume.

Further specific studies are still needed to determine the real effectiveness of CPAP, especially in healthy elderly with cardiovascular comorbidities and in patients with other risk factors for AD, to try to include the monitoring of OSA patients and provide adequate intervention even in the preventive phase of cognitive decline.

**Table 1:** Summary of the main effects of different treatments (i.e., behavioral strategies, bright light therapy, CPAP) on sleep, cognition and behaviour reported in the studies included in this review.

Treatment	Sample	Key Findings on Sleep	Key Findings on Cognition or Behaviour	Reference in the Text
**Behavioral strategies**	Cognitively healthy elderly	- Increased SWS; - Increase in sleep duration; - Improvement in sleep efficiency; - Decreased intra-sleep awakenings; - Decreased minutes awake during the night	- Decreased agitation	Naylor et al., 2012 [78]; Alessi et al., 1999 [79]; Martin et al., 2017 [80]
	MCI patients	- Improvement in sleep quality	- Improvement in processing speed domain	Wang et al., 2020 [81]
**Bright Light Therapy**	Cognitively healthy elderly	- More stable rest activity and less fragmented sleep	- Fragmented sleep and longer sleep duration were indicative of worse cognitive performance	Juda et al., 2020 [85]
	MCI	- Improvement in sleep quality and in the main circadian rhythms	- Improvement in general cognitive capabilities	Rubiño et al., 2020 [86]
	AD		- Increased agitation	Barrick et al., 2010 [87]
**CPAP**	OSA with AD	- Improvement in subjective sleep quality; - Lower percentage of N1; - Higher percentage of N2; - Decreased intra-sleep awakenings and - arousals; - Increased N3 percentage	- Improvements in the domains of: verbal episodic learning, memory and executive functions (cognitive flexibility and mental processing speed); - Slowing in cognitive decline	Ancoli-Israel et al., 2008 [93]; Cooke et al., 2009a [94]; Cooke et al., 2009b [95]; Troussière et al., 2014 [99]
	OSA without AD	- Beneficial effects on daytime sleepiness	- Improvement in executive and frontal functions and episodic verbal memory domain	Kushida et al., 2012 [102]; Rosenzweig et al., 2016 [103]

**Abbreviations:** MCI, Mild Cognitive Impairment; AD, Alzheimer’s Disease; CPAP, Continuous positive Air Pressure; OSA, Obstructive Sleep Apnoea; SWS, slow wave sleep.

## 4. The Role of NREM Sleep across the Alzheimer’s Disease Trajectory

In the last decades, the technological enhancement of research methodologies widely contributed to the characterization of the sleep alterations in the AD trajectory from the pre-clinical stage to the full-blown expression of the disease and the elucidation of their possible role in the progression of the neurodegenerative process. In this vein, studies focused on the EEG hallmarks of NREM sleep in healthy aging, MCI, and AD have had a strong impact.

### 4.1. Electrophysiological Alterations of NREM Sleep with the Progression of the AD Pathology

It is well-known that several NREM sleep EEG oscillations have an active role in memory processes and plastic mechanisms [108]. In particular, two NREM hallmarks received wider attention: slow waves and sleep spindles. Slow waves are characterized by large amplitude (>75 mV) and low frequency (0.5–4.5 Hz). NREM slow wave activity (SWA) represents a marker of sleep pressure and intensity [109], exhibiting a homeostatic [110] and local use-dependent regulation [111]. Sleep spindles are waxing-waning oscillations (11–15 Hz), generated by the interaction between thalamocortical networks and GABAergic neurons of the thalamic reticular nucleus [112]. The importance of SWA and sleep spindles for learning and plasticity is broadly accepted [108,113]. A growing number of studies points to the existence of a relationship between alterations of NREM sleep oscillations and AD progression [114]. In this vein, one line of evidence highlights signs of disrupted NREM oscillations in healthy aging and their further and specific deterioration in AD. 

Healthy aging is characterized by an alteration of both slow waves and sleep spindles, more pronounced in the frontal areas, in terms of reduced density, incidence and amplitude [115,116,117,118,119,120]. Moreover, an age-related decrease in slow waves-sleep spindles phase-locked synchrony has been recently observed [121,122]. Interestingly, the age-dependent alteration of NREM oscillations seems associated with the degree of memory impairment and grey/white matter volume integrity in the frontal regions and hippocampus [123].

AD pathology is associated with a further, progressive, and regionally-specific disruption of NREM sleep electrophysiology compared to healthy aging [114]. Beyond the consolidated evidence of SWS reduction and fragmentation in AD/MCI [124,125,126,127,128,129] associated with the severity of memory impairment [125,130], AD is characterized by greater alterations of NREM EEG hallmarks compared to normal aging, even at an early stage of the neurodegenerative disease. Indeed, SWA appears reduced along the midline in both MCI patients and healthy elderly with early signs of AD pathology [30,116,125,131]. Recent investigations were focused on the K-complex (KC), a hallmark of NREM sleep, often considered the precursor of SWA [132]. These studies showed reduced KC density in AD [126,127,129] and decreased KC amplitude in AD and MCI [129]. Interestingly, a recent longitudinal study found that KC density and amplitude decrease with time in both healthy elderly and amnesic MCI (aMCI), but the latter exhibited a greater decrease rate [133]. Moreover, with the progression of aMCI, significant differences in the KC phenomenology were observed between healthy and pathological groups, while no difference was observed at the early MCI stage [133]. A reduction of sleep spindle activity has also been observed in AD and MCI compared to healthy elderly [125,129,134,135], mainly in terms of decreased centro-parietal fast (13–16 Hz) spindle density [125,135]. Moreover, such centro-parietal reduction of sleep spindles was higher in patients with full-blown AD than MCI subjects [130]. Interestingly, both decreased KC and sleep spindles activity were associated with the degree of cognitive impairment [126,127,129,133,135]. Recently, a cross-sectional evaluation in patients with isolated subjective cognitive complaints or MCI showed that the level of cognitive decline was associated with reduced delta, theta, sigma power and maximal spindle amplitude in NREM sleep, while alterations of spindle features, self-reported sleep complaints and sleep consolidation were associated with cognitive impairment at 1-year follow-up [136].

It is worth noting that a recent study [137] has been designed to assess the relationship between the EEG features of resting wakefulness (pre- and post-sleep) and sleep stages in a large cohort of AD, MCI, and healthy elderly. The main findings show (a) a posterior reduction of sigma activity characterize NREM sleep in AD and MCI, mirroring the sleep spindles loss, (b) an EEG slowing can be observed in both wakefulness and REM sleep in AD/MCI with strong correlations between the two phenomena, suggesting a common neuropathological mechanism, (c) the analysis of evening-to-morning modifications in waking EEG points to a progressive disappearance in AD and MCI of overnight changes in delta activity, suggesting a gradual decay of the sleep restorative function on diurnal EEG activity with the worsening of the disease, (d) such phenomenon is correlated with the impairment of high-frequency activity during NREM and REM sleep in AD.

Overall, these results highlight that (a) healthy aging is characterized by a disruption of several NREM sleep hallmarks; (b) such NREM deterioration is progressively greater and locally-specific with the development of the AD pathology; (c) the alteration of NREM oscillations is associated with the level of cognitive decline; (d) early disruption of different NREM sleep oscillations, even during the pre-clinical stage of AD, can predict the development of the neurodegenerative process; (e) a complex relationship characterizes inter-dependent wakefulness and sleep electrophysiological modifications in AD and MCI. 

It is worth noting that many investigations also found macro- and microstructural modifications of REM sleep in AD/MCI [13,125,138,139,140]. However, the main sleep EEG-based therapeutic strategies proposed in the present literature (see below) are focused on the modulation of NREM oscillations. Therefore, a detailed dissertation about REM sleep abnormalities in AD goes beyond the aims of the present review.

### 4.2. A Mechanistic Link between NREM Sleep Alterations and AD Progression

The reviewed findings raise the possibility that NREM sleep alterations may represent not only a marker of AD but also a risk factor of its evolution. Hence, a specific mechanistic relation may link the alteration of NREM sleep oscillatory events and the pathophysiology of AD. In line with this hypothesis, many animal and human researches have been designed to describe the possible relation between NREM sleep disruption and early markers of AD. Starting from the notion that Aβ and tau pathology start their accumulation before the appearance of the cognitive impairment associated with AD [141], the research in this field has been conducted not only in full-blown AD but it has been widely extended to healthy aging. Indeed, the existence of a possible relationship between sleep disruption and signs of AD pathology before the beginning of cognitive deterioration would imply that specific sleep alterations may represent early biomarkers of the neurodegenerative process. At present, many findings go in this direction [10,114,142].

In a pioneering study, Kang and co-workers [143] showed in the AD mouse model that brain interstitial fluid levels of Aβ were regulated by the sleep-wake cycle, showing an increase with time spent awake. More recently, findings from Xie and co-workers [32] pointed to a crucial role of sleep in neurotoxic waste products clearance. Specifically, they observed that SWS in mice enhanced Aβ clearance compared to wakefulness. Taken together, these results highlight a direct and reciprocal link between the level of Aβ burden, the wake-sleep cycle, and the functions of SWS. Moreover, a large body of evidence shows that different measures of sleep disruption are associated with AD biomarkers (i.e., Aβ, phosphorylated tau, neurodegeneration) in animals [144,145,146] and humans [13,30,130,147,148,149,150,151,152,153,154]. The relation between sleep and Aβ received wider attention, probably due to findings associated with the “amyloid cascade hypothesis”, which proposes the Aβ burden as the main trigger of AD pathology [10]. However, evidence about the association between sleep disruption and other AD hallmarks is growing.

In this context, the existence of a specific relation between NREM sleep alterations and AD pathology has received strong empirical support. Mice overexpressing Aβ protein exhibited reduced and more fragmented NREM sleep [143,144]. Mander and coworkers [149] found that impaired NREM SWA was associated with Aβ burden in the medial prefrontal cortex and weakened memory consolidation in cognitively normal older humans. Moreover, signs of decreased and fragmented SWS in cognitively normal elderly was associated with higher cerebrospinal fluid (CSF) level of Aβ42 [30], and SWA was found negatively correlated with CSF Aβ in middle-aged [155] and older adults [30]. Decreased NREM SWA, particularly in its slowest frequencies, has been observed in the presence of Aβ deposition and tau accumulation in predominantly cognitively normal subjects [131], and Aβ burden was found associated with reduced amplitude of <1 Hz SWA [154]. In line with these findings, it has been observed that both acute and chronic sleep deprivation can increase levels of Aβ and tau pathology in humans and animals [143,156,157,158,159,160,161,162,163,164]. Crucially, selective SWA disruption in healthy adults was associated with increased Aβ [69]. This effect was specific for SWA (and not for sleep efficiency and duration) and Aβ (and not total protein, tau, YKL-40, or hypocretin) [69]. Finally, longitudinal evaluation confirmed that sleep disruption is associated with future AD-related outcomes [22,165,166,167,168,169], and the proportion of NREM SWA <1 Hz and sleep efficiency in older adults can selectively predict the speed of subsequent deposition of Aβ over time [170].

Overall, cross-sectional studies, sleep deprivation/disruption protocols, and longitudinal evaluations support the notion of a mechanistic link between NREM sleep impairment and AD pathology, with wider evidence about SWA and Aβ burden. In this vein, an extensively accepted hypothesis concerns the existence of a bidirectional relation between these phenomena: NREM sleep alterations, particularly concerning SWA, would be associated with greater expression of AD pathology, which in turn induces a further impairment in NREM sleep oscillations [142,171]. According to this model, SWS in healthy subjects normally compensates for the diurnal wakefulness-related Aβ accumulation, promoting a decrease of the Aβ burden. The reduction of SWA in healthy aging, even more evident in MCI and AD, should be associated with an impairment of the SWS-related Aβ clearance, which in turn has a negative effect on SWA, generating a vicious cycle that promotes the progression of the AD neurodegenerative process [142,171]. However, a causal relationship between sleep deterioration and the emergence of AD biomarkers has not been directly established.

Beyond SWA, the possible relation between sleep spindle deterioration and markers of AD pathology has also been investigated. A recent study showed that impaired SO-spindle coupling in older adults predicted a greater tau burden in the medial temporal lobe [154]. Interestingly, such association with SO-spindles coupling was specific for tau accumulation: no relation was observed between SO-spindles coupling with Aβ burden, which instead predicted the decreased amplitude of <1 Hz SWA [154]. This result may suggest the existence of separate and unique associations between different sleep-EEG features (i.e., SO-spindle coupling, SWA) and specific markers of AD pathology (i.e., tau, Aβ). Moreover, N2 sleep spindles features in older adults were negatively correlated to CSF T-tau levels and, secondly, P-tau and Aβ42, while no relation was observed between CSF tau and SWA [128]. Albeit the relationship between sleep spindles and tau-pathology needs of further investigations, the authors proposed that (a) spindles generation may be negatively affected by tau accumulation, probably in the brainstem cells that project to the thalamic reticular nucleus, (b) sleep spindles may weaken the production or enhance the metabolism of tau, (c) changes in spindle density and tau levels may represent common consequences of an upstream mechanism [128].

According to Mander [114], the available findings on the sleep-AD relation suggest that the pathological components of AD (i.e., Aβ, tau, neurodegeneration) are specifically associated with alterations in local and global expressions of sleep depending on the cerebral area affected. Such sleep alterations evolve with the progression of the neurodegenerative process and may be related to memory impairment.

## 5. Innovative NREM Sleep-Based Techniques for Prevention and Care of Alzheimer’s Disease

The hypothesis of a bidirectional influence between NREM sleep alterations and AD pathological components implies that therapeutic strategies aimed to improve NREM sleep may represent innovative solutions to contrast the development of AD. According to the proposed literature, the rationale would be that the improvement of NREM sleep oscillations should promote Aβ clearance, decreasing neurotoxic waste accumulation and oxidative stress, and thus improving memory performance [142]. Consistently, the improvement of cortical SOs through optogenetic stimulation in rodents induces a reduction in the formation of Aβ plaques [172]. Therefore, the direct modulation of NREM sleep electrophysiology may be a promising therapeutic strategy, with different aims for specific stages of the AD trajectory.

A first hypothetical objective of NREM sleep enhancement would be the prevention of AD pathology. The strategic modulation of local sleep oscillations may be used in healthy aging and in the pre-clinical stage of AD to reinforce the protective role of NREM sleep against the pathological markers of AD, thus preventing the neurodegenerative process. On the other hand, the enhancement of specific sleep oscillations may potentially have a role in the treatment of full-blown AD, slowing down the progression of the neurodegeneration through the contrast of the vicious cycle between NREM alterations and AD expression.

Beyond pharmacological treatments, several non-invasive techniques have recently demonstrated their ability to modulate the human EEG oscillations during sleep with beneficial effects on memory [173,174]. Therefore, the interest in their possible application in healthy and pathological aging is progressively growing [175,176,177], and several encouraging results are available (Table 2). This approach fits with the general notion that monitoring and managing sleep EEG features may support brain plasticity-dependent process during rehabilitative paradigms in different pathological conditions [178].

### 5.1. Transcranial Current Stimulation

Promising findings came from research on transcranial current stimulation (tCS) protocols, a group of brain stimulation techniques designed to non-invasively affect cerebral physiology and cognitive processes [179]. Such methods involve the delivery of low-intensity current through the skull by at least two surface electrodes. Different behavioural and physiological modifications can be obtained depending on the specific stimulation parameters, such as stimulation site, location of the reference, duration, waveform, frequency, polarity, and intensity. A growing body of evidence shows that tCS techniques can induce changes in sleep electrophysiology [180] and several findings suggest that these procedures may represent valid candidates to bidirectionally affect sleepiness levels and the electrophysiological processes that characterize sleep onset [180,181]. Crucially, several tCS protocols applied during sleep can enhance NREM sleep oscillations and, in turn, promote memory consolidation in healthy subjects [182,183,184,185,186]. Moreover, similar results have also been observed in clinical conditions like schizophrenia [187] and attention-deficit/hyperactivity disorder [188]. Albeit some findings did not replicate these results [189,190], a recent meta-analysis concludes that tCS during sleep can enhance memory consolidation in the declarative domain [191]. Starting from these findings, several studies tried to apply tCS techniques to the elderly population. 

The first study in this field used a bi-frontal oscillating anodal transcranial direct current stimulation (tDCS) protocol in the slow oscillations (SOs) frequency (0.75 Hz; so-tDCS) applied during nocturnal NREM sleep in healthy elderly adults [192]. The authors found no effect of the stimulation on sleep-dependent declarative and procedural memory consolidation, while increased time awake and reduced NREM stage 3 sleep were observed. In contrast, Westerberg and coworkers [193] showed the efficacy of the so-tDCS protocol (compared to a sham stimulation) delivered during an afternoon nap to selectively improve declarative memory consolidation in healthy older adults, accompanied by increased frontal SO activity and reduced central fast spindle density. No changes in SWS duration and subjective sleep quality were found. Although EEG changes and learning performance were not directly related, this study represents the first demonstration of the possibility of improving both NREM electrophysiology and memory consolidation in an elderly population with tCS protocols.

Partially consistent results have been found by Ladenbauer and coworkers [194]. Indeed, so-tDCS during a nap improved picture memory retention and increased frontal SO activity and fast spindle activity in older adults compared to a sham protocol, without changes in sleep architecture and spatial and verbal memory. Again, sleep EEG changes were not directly associated with behavioural outcomes.

The same stimulation protocol has been used in healthy elderly during the first NREM period of night sleep [195]. Compared to sham, so-tDCS increased SWA and spindle activity immediately after the stimulation, but visuo-spatial memory consolidation was unexpectedly impaired, without verbal and procedural memory changes. Concerning the sleep architecture, NREM sleep stage 4 was reduced and time awake after sleep onset was (not significantly) increased. 

Only one study assessed the effect of so-tDCS compared to sham during a daytime nap in subjects with aMCI [196]. Consistently with the administration of an identical protocol in healthy elderly [194], the stimulation induced an improvement in visual declarative memory but not in spatial and verbal memory. Considering the EEG measures, so-tDCS provoked an increase in SO and spindle power and enhanced SO-fast spindles power coupling. Moreover, the only active stimulation effect observed on sleep architecture was an increase in stage 2. Interestingly, performance in visual memory was associated with the greater SO-fast spindles coupling but not with changes in overall SO and spindle power. 

Overall, results about the administration of so-tDCS during sleep in healthy elderly and subjects with MCI to promote NREM sleep oscillations and memory consolidation appear promising, albeit not uniform. Beyond several methodological differences between the reviewed studies that may have affected the results (e.g., time of stimulation, electrodes’ position; presence/absence of ramping procedures at the beginning and the end of the stimulation; sample homogeneity; type of stimuli), it should be noted that the active stimulation had a negative influence on sleep architecture in both studies that found no differences or detrimental effects on memory performance compared to sham [193,196]. Therefore, it is possible that this phenomenon may have prevented the beneficial effect of the stimulation on memory consolidation. In line with this hypothesis, it has been proposed that age-adjusted tCS protocols should be developed for the nocturnal administration on elderly subjects [195].

### 5.2. Auditory Stimulation

Another approach for the modulation of NREM sleep oscillations to enhance cognitive functioning is represented by auditory stimulation. The delivery of specific types of acoustic stimuli during sleep can affect NREM oscillations [197,198,199,200]. Moreover, novel stimulation methodologies allow to automatically deliver an auditory cue when a specific phase of an endogenous oscillatory event is detected during sleep [173]. In this vein, the application of auditory tones phase-locked to the down-to-up phase transition of a SO can enhance low-frequency EEG activity (SWA power; SOs), spindle activity and memory performance [201,202,203,204,205,206]. Therefore, several authors recently applied this strategy in the elderly population with the aim to boost NREM electrophysiological hallmarks and improve memory [177]. 

In the first study, Papalambros and coworkers [207] assessed the effects of acoustic stimulation phase-locked to the up-state of the slow waves during nocturnal sleep in healthy older subjects. The authors found that the auditory stimulation improved overnight declarative memory performance compared to a sham condition, without changes in sleep architecture and subjective sleep quality. While the active stimulation did not differ from the sham condition concerning the SWA during the entire night, it increased SWA and spindle activity during the stimulation blocks. The enhancement of SWA was associated with overnight memory improvement.

In contrast with these encouraging results, Schneider and coworkers [208] recently found an impaired declarative memory retention after acoustic close-loop stimulation during a night of sleep in healthy middle-aged and older adults, without changes in the procedural domain and post-sleep encoding. No stimulation-induced effect was found on sleep architecture. From an electrophysiological standpoint, the stimulation prolonged endogenous trains of SOs and induced sleep spindles phase-locked to the SO up-states. However, a direct comparison with a younger cohort showed in the older subject (a) a reduction of the brain responses and (b) a different temporal dynamic of the stimulation effects on SO and sleep spindles.

Finally, only one study assessed the effects of acoustic stimulation phase-locked to the upstate of the sleep slow waves in a small sample of aMCI patients, compared to a sham condition [209]. The authors found an increase in SO activity and SWA during the stimulation intervals compared to sham, associated with enhanced morning word recall. However, only five out of nine patients showed an increased memory recall, resulting in the absence of significant differences in behavioural performance between stimulation and sham. No changes were observed in sleep spindles and sleep architecture. 

Taken together, the few studies on phase-locked loop acoustic stimulation in the elderly to modulate sleep physiology and enhance memory reveal encouraging but heterogeneous results. Interestingly, recent findings suggest that closed-loop auditory stimulation applied to enhance SOs may be more effective during specific phase windows, which would be different in younger and older adults [210]. In this view, future researches should be focused on the assessment of the optimal stimulation’s parameters in healthy and pathological aging.

### 5.3. General Considerations

The proposed application of strategies to enhance NREM sleep oscillations with the aim to prevent or slow down AD pathology has a solid scientific background, and findings in this field appear promising. However, we are far from the possibility to employ these strategies in a clinical context. The number of studies is still scarce, and results are heterogeneous. Moreover, beyond specific methodological points, partially explaining such outcome variability that should be controlled in future studies [177], several issues should be considered.

Concerning the possibility to use these strategies in healthy subjects to prevent AD development, the observation of their ability to enhance NREM oscillations and memory after one night does not constitute sufficient evidence. Indeed, their long-term influence on sleep physiology and memory and their direct efficacy in preventing AD symptoms are still unknown. A ceiling effect associated with prolonged stimulation is conceivable. For these reasons, the efficacy of these methods during extended periods of time needs to be tested. The same issue can be raised concerning the possibility to use these techniques to slow down the neurodegenerative process in full-blown AD. Moreover, studies on the direct application of these strategies in AD patients are still missing. A further effort is needed to provide concrete evidence about the efficacy of these strategies along the different stages of the AD trajectory.

It is worth noting that the reviewed strategies (i.e., tCS and auditory stimulation) are not the only techniques to modulate NREM sleep electrophysiology. Indeed, we considered only those methods with consolidated literature in young adults and several available results in the aging population. However, other strategies may be of potential interest in this field. Transcranial magnetic stimulation (TMS) applied during sleep can modulate SWA when applied in healthy young adults [211]. However, while the modulatory effects of TMS are topographically accurate [212,213], its demanding setup actually reduces the possibility of a nocturnal application outside of an experimental or clinical laboratory. On the other hand, recent studies suggest that NREM sleep oscillations can be enhanced by rocking stimulation [214,215,216], with a beneficial effect on memory consolidation [216]. If the potential beneficial effect of this strategy on sleep physiology and memory processes will be confirmed, their investigation in healthy and pathological aging should be considered. Actually, the only recent study on the application of gentle rocking movements during sleep in healthy elderly showed no effects on memory performance and a reduction in delta power [217]. Hence, the efficacy of this technique in this population needs further assessment.

## 6. Conclusions

The development of early intervention strategies aimed to reduce AD risk and slow down the neurodegenerative process represents a great challenge in current research and clinic. In this field, the agreement about the view of sleep as a crucial target for therapeutic intervention is progressively growing, thanks to a large number of studies pointing to (a) the existence of strong and progressive sleep alterations in the AD pathology, (b) the predictive role of early sleep dysfunctions on subsequent cognitive decline, (c) the bidirectional relationship between sleep alterations (particularly concerning NREM sleep oscillations) and AD [10,142]. Accordingly, the reviewed findings highlight the importance of sleep monitoring and promotion in healthy aging to prevent the development of AD. Moreover, they suggest that sleep-based therapeutic approaches can positively impact the cognitive status in different stages of the AD pathology. We reviewed the evidence about the efficacy of different types of non-pharmacological interventions on sleep alterations and disorders in healthy and pathological aging, albeit the timing of these interventions and their impact on the cognitive status needs further assessment. Finally, several techniques seem able to promote NREM sleep oscillations and memory performance. However, we are still far from the possibility to use techniques to directly modulate AD-related sleep EEG features with a clinical purpose. Results are promising, but a further effort to overcome the present methodological limitations is needed. 

## Figures and Tables

**Table 2:** Main features and key findings of studies reporting data on the effect of transcranial current stimulation and auditory stimulation techniques on NREM sleep features and memory in healthy and pathological aging.

Reference	Sample	Age	Stimulation Parameters	Key Findings on Sleep	Key Findings on Memory
**Transcranial current stimulation**					
Eggert et al., 2013	26 cognitively healthy older adults (10 M)	Mean: 69.1 y Range: 60–90 y	Type: anodal sinusoidally oscillating stimulation Period: early NREM during night sleep Duration: 31:20 min (five intervals, each composed of 5:16 min of stimulation and 1 min free of stimulation) Frequency: 0.75 Hz Site: bilateral frontal (F3-F4) locations, referenced to the mastoids	- Increased time awake and reduced NREM stage 3 in the five 1-min stimulation free intervals	- Absence of effects on memory
Westerberg et al., 2015	19 cognitively healthy older adults (3 M)	Mean: 73.4 y Range: 65–85 y	Type: anodal sinusoidally oscillating stimulation Period: afternoon nap, starting 4 min after the onset of stage 2 Duration: 30 min (five alternating 5-min “on” and 1-min “off periods) Frequency: 0.75 Hz Site: bilateral frontal (F7-F8) locations, referenced to the mastoids	- Increased frontal SO activity - Reduced central fast spindle density	- Improvement of verbal recall
Landebauer et al., 2016	18 healthy older subjects (8 M)	Mean: 65 Range: 57–77 y	Type: anodal sinusoidally oscillating stimulation Period: afternoon nap, starting 4 min after the onset of stable stage 2 Duration: five 5-min blocks of stimulation separated by 1:40 min of stimulation-free inter-block intervals Frequency: 0.75 Hz Site: bilateral frontal (F3-F4) locations, referenced to the mastoids	- Increased frontal SO activity - Increased frontal and parietal fast spindle activity	- Improvement of picture memory retention
Paßmann et al., 2016	21 healthy older adults (11 M)	Mean: 65 y	Type: anodal sinusoidally oscillating stimulation Period: early NREM during night sleep Duration: five 5-min blocks of stimulation separated by 1 min of stimulation-free inter-block intervals Frequency: 0.75 Hz Site: bilateral frontal (F3-F4) locations, referenced to the mastoids	- Increased power in SO activity and spindle frequency bands - Reduced NREM Stage 4 sleep for the entire night and (non-significant) increase of time awake after sleep onset	- Decreased visuo-spatial performance
Landebauer et al., 2017	16 aMCI patients (9 M)	Mean: 71 y Range: 53–81 y	Type: anodal sinusoidally oscillating stimulation Period: afternoon nap, starting 4 min after the onset of stable stage 2 Duration: five 5-min blocks of stimulation separated by 1:40 min of stimulation-free inter-block intervals Frequency: 0.75 Hz Site: bilateral frontal (F3-F4) locations, referenced to the mastoids	- Increased SO and sleep spindle power - Enhanced SO-fast spindle coupling - Increased NREM Stage 2 sleep	- Improved visual memory performance - Association between visual memory performance and greater SO-fast spindle coupling
**Auditory stimulation**					
Papalambros et al., 2017	13 cognitively healthy older adults (3 M)	Mean: 75.2 y Range: 60–84 y	Type: phase-locked acoustic stimulation; slow wave detection Period: NREM of entire night sleep Stimulus: Pink noise (30–50 db) Protocol: 50 ms pulses at ~0.85 Hz (adaptive) in blocks of 5, separated by ~1.2 s, followed by ~6 s off period.Target phase:20 degree to slow-wave peak	- Increased SWA and spindle density and amplitude during the stimulation blocks	- Improvement of overnight declarative memory performance - Overnight memory improvement associated with the enhancement of SWA
Schneider et al., 2020	17 healthy middle-aged adults (8 M)	Mean: 55.7 y Range: 49–63 y	Type: auditory closed loop stimulation; slow wave detection Period: 3.5 h from the first stable NREM sleep Stimulus: Pink noise (mean: 54.5 dB) Protocol: two pulses separated by and individual delay (mean 1091.47 ms), followed by a detection pause of 2.5 s	- Prolonged endogenous train od SOs - Increased fast sleep spindles activity phase-locked to the induced SO up-states - Compared to a group of younger cohort, the older adults showed (a) reduction of the brain responses, and (b) different temporal dynamics of the stimulation effect on SO and sleep spindles	- Impaired declarative memory retention
Papalambros et al., 2019	9 aMCI patients (4 M)	Mean: 72 y Range: 62-86 y	Type: phase-locked acoustic stimulation; slow wave detection Period: NREM of entire night sleep Stimulus: Pink noise (30–50 db) Protocol: 50 ms pulses at ~0.85 Hz (adaptive) in blocks of 5, separated by ~1.2 s, followed by ~6 s off period.Target phase: 20 degree to slow-wave peak	- Increase in SO activity and SWA during the stimulation intervals	- Increased word-pair recall in only five out of nine patients. - Enhanced word-pair recall associated with increased SWA

**Abbreviations:** M, males; NREM, non-rapid eye movement; SO, slow oscillation; SWA, slow wave activity; y, years.

## Data Availability

Not applicable.
